# Discontinuation of methotrexate in rheumatoid arthritis patients achieving clinical remission by treatment with upadacitinib plus methotrexate (DOPPLER study): A study protocol for an interventional, multicenter, open-label and single-arm clinical trial with clinical, ultrasound and biomarker assessments

**DOI:** 10.1097/MD.0000000000028463

**Published:** 2022-01-14

**Authors:** Toshimasa Shimizu, Shin-ya Kawashiri, Shuntaro Sato, Yurika Kawazoe, Shohei Kuroda, Rina Kawasaki, Yasuko Ito, Shimpei Morimoto, Hiroshi Yamamoto, Atsushi Kawakami

**Affiliations:** a Clinical Research Center, Nagasaki University Hospital, Nagasaki, Japan; b Departments of Immunology and Rheumatology, Division of Advanced Preventive Medical Sciences, Nagasaki University Graduate School of Biomedical Sciences, Nagasaki, Japan; c Community Medicine and, Division of Advanced Preventive Medical Sciences, Nagasaki University Graduate School of Biomedical Sciences, Nagasaki, Japan; d Innovation Platform & Office for Precision Medicine, Nagasaki University Graduate School of Biomedical Sciences, Nagasaki, Japan.

**Keywords:** biomarker, Janus kinase inhibitor, musculoskeletal ultrasound, rheumatoid arthritis, upadacitinib

## Abstract

**Background:**

The administration of Janus kinase inhibitors as well as biological disease-modifying anti-rheumatic drugs has dramatically improved the clinical outcomes of patients with rheumatoid arthritis (RA). Previous trials have shown that upadacitinib, a Janus kinase inhibitor, can effectively improve disease activity and prevent progression of joint destruction in RA patients with inadequate responses to methotrexate (MTX). It remains unclear whether reduced disease activity can be maintained after discontinuation of MTX in patients treated with upadacitinib plus MTX. Thus, the aim of this study is to evaluate changes in disease activity after administration of upadacitinib plus MTX in RA patients who failed to achieve an adequate response to MTX and to determine whether clinical relapse can be avoided after discontinuation of MTX in those who achieved clinical remission.

**Methods/design:**

The proposed study is an interventional, multicenter, open-label, single-arm clinical trial with a 48-week follow-up. The cohort will include 155 RA patients with at least moderate disease activity during treatment with MTX. Patients will receive upadacitinib and MTX will be discontinued for those who achieve clinical remission. Disease activity will be evaluated longitudinally by measuring clinical disease activity indices and with musculoskeletal ultrasound (MSUS). The primary endpoint is the proportion of patients who sustain a disease activity score-28- C reactive protein score of ≤3.2 from week 24 to 48 after a disease activity score-28- C reactive protein score of <2.6 at week 24. Important secondary endpoints are changes from baseline MSUS scores. Serum levels of multiple biomarkers, including cytokines and chemokines, will be comprehensively analyzed.

**Discussion:**

The study results are expected to show the clinical benefit of the discontinuation of MTX after achieving clinical remission by treatment with upadacitinib plus MTX combination therapy. The strength of this study is the prospective evaluation of therapeutic efficacy using clinical disease activity indices and standardized MSUS, which can accurately and objectively evaluate disease activity at the joint level among patients drawn from multiple centers. Furthermore, parameters to predict clinical remission after administration of upadacitinib plus MTX combination therapy and nonclinical relapse after discontinuation of MTX will be screened by integrated multilateral assessments (i.e., clinical disease activity indices, MSUS findings, and serum biomarkers).

## 1 Introduction

Rheumatoid arthritis (RA) is a chronic autoimmune disease that causes synovitis in multiple joints.^[1]^ Because uncontrolled RA can lead to joint destruction and functional disability, management of RA based on the “treat-to-target” strategy is important to control disease Activity.^[2]^ Methotrexate (MTX) is a first-choice conventional synthetic disease-modifying anti-rheumatic drug (csDMARD) for treatment of active RA. However, a considerable proportion of RA cases are refractory to MTX. In addition, adverse events (AEs) and tolerability issues may occur with long-term use of MTX. Thus, the next treatment choice is important for RA patients refractory to or intolerant of MTX. Janus kinase (JAK) inhibitors in addition to biological DMARDs (bDMARDs) have been used in the second phase of the European League Against Rheumatism (EULAR) recommendations, lading to better clinical outcomes, such as the achievement of clinical remission for patients with RA.^[3]^

Upadacitinib is a selective JAK1 inhibitor developed by AbbVie (Chicago, IL). The addition of JAK inhibitors, including upadacitinib, was reported to achieve clinical remission in nearly half of RA patients with inadequate responses to MTX.^[4,5]^ Although there is little evidence, as the next step, it is important to determine whether minimal disease activity can be maintained even if MTX is discontinued after achieving clinical remission in patients treated with a combination of JAK inhibitors and MTX. Thus, it is necessary to investigate the maintenance of clinical non-relapse after discontinuation of MTX in RA patients who achieved clinical remission with upadacitinib plus MTX combination therapy.

Musculoskeletal ultrasound (MSUS) is widely applied as an imaging modality for clinical assessment of RA patients.^[6,7]^ MSUS provides more accurate detection of synovial inflammation as compared to a clinical examination. In addition, MSUS is noninvasive, objective, relatively inexpensive, repeatable, and thus well-suited for treatment monitoring.^[6,7]^ It is therefore important to assess disease activity and therapeutic response using MSUS as well as the clinical disease activity indices, including subjective parameters.^[6–9]^ However, very few prospective multicenter collaborative studies have used standardized MSUS to evaluate the disease activity of RA.

We will evaluate the changes in disease activity by using MSUS as well as clinical disease activity indices so that the patients’ disease activity will be assessed with greater accuracy. In addition, MSUS will be used to determine whether nonclinical relapse can be maintained after discontinuation of MTX by RA patients who achieved clinical remission. Furthermore, serum biomarker levels will also comprehensively analyzed. Lastly, patient parameters will be screened to predict nonclinical relapse after discontinuation of MTX by integrating multilateral assessments, including the clinical disease activity indices, MSUS findings, and serum biomarkers.

This clinical trial was named the “Discontinuation of methotrexate in rheumatoid arthritis patients achieving clinical remission by treatment with upadacitinib plus methotrexate (DOPPLER)” study. Herein, the final protocol (version 1.5; July 27, 2021) of the DOPPLER study is described.

## 2 Objectives

### 2.1 Primary objective

The principal objective of the study is to determine the proportion of RA patients who maintained nonclinical relapse after discontinuation of MTX after achieving clinical remission with upadacitinib plus MTX combination therapy.

### 2.2 Secondary objectives

Following upadacitinib administration, disease activity will be assessed by MSUS. We will explore whether patients’ baseline parameters, including clinical disease activity indices, MSUS scores, and serum biomarkers, can predict a clinical response after the administration of upadacitinib. In addition, we will explore whether patients’ parameters, including clinical disease activity indices, MSUS scores, and serum biomarkers at the discontinuation of MTX, can predict a nonclinical relapse after the discontinuation of upadacitinib.

## 3 Methods/design

### 3.1 Study design

The study was designed in accordance with the Standard Protocol Items: Recommendations for Interventional Trials and Consolidated Standards of Reporting Trials 2010 guidelines^[7,8]^ (Additional File 1). The proposed study is a prospective, open-label, single-arm, and interventional clinical trial that will be conducted at the following 53 centers: Nagasaki University Hospital, Hokkaido University Hospital, Hokkaido Medical Center for Rheumatic Diseases, Katayama Orthopedic Rheumatology Clinic, University of Tsukuba Hospital, Saitama Medical University Hospital, Aoki Medical Clinic, Chiba University Hospital, Yokohama City University Medical Center, Yokohama City University Hospital, Yokohama Minami Kyousai Hospital, Yoshimi Hospital, Toyota Kosei Hospital, Chubu Rosai Hospital, Kyoto University Hospital, University Hospital Kyoto Prefectural University of Medicine, Osaka Medical and Pharmaceutical University Hospital, Osaka City University Hospital, National Hospital Organization Osaka Minami Medical Center, Kindai University Hospital, Kita-Harima Medical Center, Kobe University Hospital, Okayama University Hospital, Yamaguchi Prefectural Welfare Agricultural Cooperative Association Nagato General Hospital, Kagawa University Hospital, Ehime University Hospital, Kochi Medical School Hospital, Hospital of the University of Occupational and Environmental Health, Tobata General Hospital, PS Clinic, National Hospital Organization Ureshino Medical Center, National Hospital Organization Miyakonojo Medical Center, Miyazaki Zenjinkai Hospital, Chiyoda Hospital, Yoshitama Clinic for Rheumatic Diseases, Eiraku Clinic, Japanese Red Cross Nagasaki Genbaku Hospital, National Hospital Organization Nagasaki Medical Center, Sasebo Chuo Hospital, Sasebo City General Hospital, Fukushima Medical University Hospital, Toho University Ohashi Medical Center, Niigata Rheumatic Center, Nara Medical University Hospital, Tohoku Medical and Pharmaceutical University Wakabayashi Hospital, Aomori Prefectural Central Hospital, Nippon Medical School Hospital, Kurume University Medical Center, Seirei Hamamatsu General Hospital, Marunouchi Hospital, St. Marianna University Hospital, Japanese Red Cross Ogawa Hospital, and Shinonoi General Hospital.

**Figure 1:**
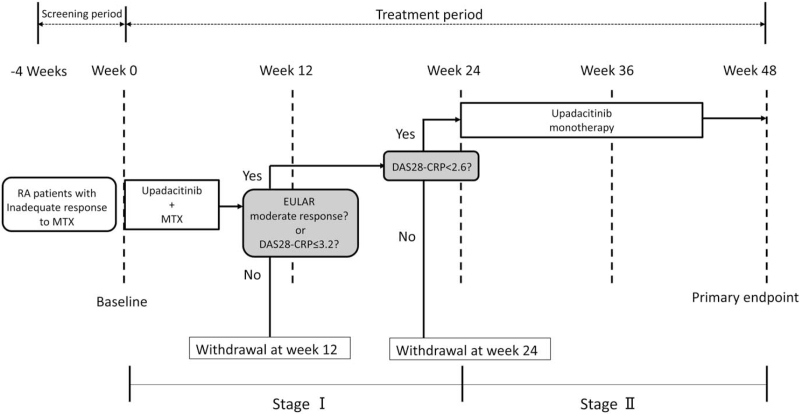
Study design.

In total, 155 RA patients will receive upadacitinib for a duration of 48 weeks. The study design is summarized in Figure 1.

### 3.2 Approvals

The study protocol was approved by the certified review board (CRB) of Nagasaki University (CRB approval no.: CRB20-024) and is registered with the Japan Registry of Clinical Trials (https://jrct.niph.go.jp) as jRCTs071200079 and ClinicalTrials.gov (https://clinicaltrials.gov/) as NCT05121298. The study will be conducted in accordance with the study protocol, the principles of the Declaration of Helsinki, the Clinical Trials Act (Act No. 16 of April 14, 2017), the Act on the Protection of Personal Information, and all other pertinent regulations. Participants will be provided with an explanation regarding the study by their treating rheumatologist and asked to voluntarily sign an informed consent form before participation.

### 3.3 Participants

#### 3.3.1 Inclusion criteria

Patients must meet all of the following requirements prior to consideration for study inclusion: (1) age ≥20 years; (2) diagnosis of RA based on the American College of Rheumatology/EULAR 2010 RA Classification Criteria^[10]^; (3) at least moderate disease activity defined as a Disease Activity Score 28 (DAS28)-C reactive protein (CRP) score of >3.2 at the time of eligibility evaluation; (4) at least one power Doppler (PD)-positive joint of 22 joints examined by MSUS at the time of eligibility evaluation, (5) treatment with MTX for ≥8 weeks prior to providing consent, including 4 weeks or more at the same dosage of 6–16 mg per week; and (6) ability and willingness to provide written informed consent and comply with the requirements of the study protocol.

#### 3.3.2 Exclusion criteria

The exclusion criteria are as follows: (1) concurrent use of a corticosteroid equivalent to >7.5 mg/d of prednisolone; (2) applicable an item for the contraindication of upadacitinib (3) previous use of a JAK inhibitor; (4) treatment with a corticosteroid and change in dosage within 4 weeks prior to providing consent; (5) treatment with a csDMARD, other than MTX, within 2 weeks prior to providing consent; (6) treatment with a biologic DMARD or a biosimilar DMARD (i.e., infliximab, biosimilar of infliximab, adalimumab, golimumab, certolizumab pegol, tocilizumab, sarilumab, or abatacept) within 8 weeks prior to providing consent; (7) treatment with a tumor necrosis factor (TNF) inhibitor (i.e., etanercept or biosimilar of etanercept) within 4 weeks prior to providing consent; (8) use of a prohibited drug or therapy, other than the agents noted above, within 4 weeks prior to providing consent; (9) any complication, other than RA, causing a musculoskeletal disorder (i.e., ankylosing spondylarthritis, reactive arthritis, psoriatic arthritis, crystal-induced arthritis, systemic lupus erythematosus, systemic scleroderma, inflammatory myopathy, or mixed connective tissue disease); (10) current pregnancy, breastfeeding, or noncompliant with a medically approved contraceptive regimen during and 12 months after the study period; or (11) inappropriateness for inclusion in this study as determined by the investigators.

#### 3.3.3 Intervention

Patients will receive upadacitinib at 15 mg/day and continue to receive the same dosage of MTX with or without a corticosteroid for 24 weeks (Stage I). If a EULAR-defined moderate response or a DAS28-CRP score of ≤3.2 is achieved at 12 weeks with a DAS28-CRP score of <2.6 at 24 weeks, MTX will be discontinued and upadacitinib with or without a corticosteroid will be continued until week 48 (Stage II). Patients not achieving a EULAR-defined moderate response and a DAS28-CRP score of ≤3.2 at 12 weeks or <2.6 at 24 weeks will be excluded from this study.

During the study period, the following treatments are prohibited: administration of a bDMARD or JAK inhibitor, other than upadacitinib; concomitant use of an immunosuppressant (i.e., azathioprine, cyclophosphamide, or cyclosporine), csDMARD, other than MTX, or an oral corticosteroid equivalent to >7.5 mg/day of prednisolone; intra-articular corticosteroid injections at the joints; and any nonsteroidal anti-inflammatory drug suppository. During the study period, the dosage of any oral nonsteroidal anti-inflammatory drug can be modified within the range approved in Japan.

#### 3.3.4 Patient discontinuation criteria

A patient may be prematurely withdrawn from the study for the following reasons:

- Discontinuation of upadacitinib for more than 7 consecutive days
- Failure to achieve a EULAR-defined moderate response and a DAS28-CRP score of ≤3.2 at week 12
- Failure to achieve a DAS28-CRP score of <2.6 at week 24
- Discontinuation of MTX for more than 2 consecutive weeks during Stage I
- Any clinical relapse, defined as an increase in a DAS28- erythrocyte sedimentation rate (ESR) score of ≥3.2 due to elevated disease activity of RA, after discontinuation of MTX
- Patient request to quit the trial
- Patient request to change or discontinue treatment
- Any AE inhibiting continued participation
- Pregnancy
- Discretion of the principal investigator to discontinue trial participation to ensure patient safety

### 3.4 Outcome measurements

Study visits will be conducted at baseline and 12, 24, 36, and 48 weeks after initiation of upadacitinib administration. The assessments are presented in Table 1. Clinical physicians will be blinded to joint assessments by MSUS.

**Table 1:**
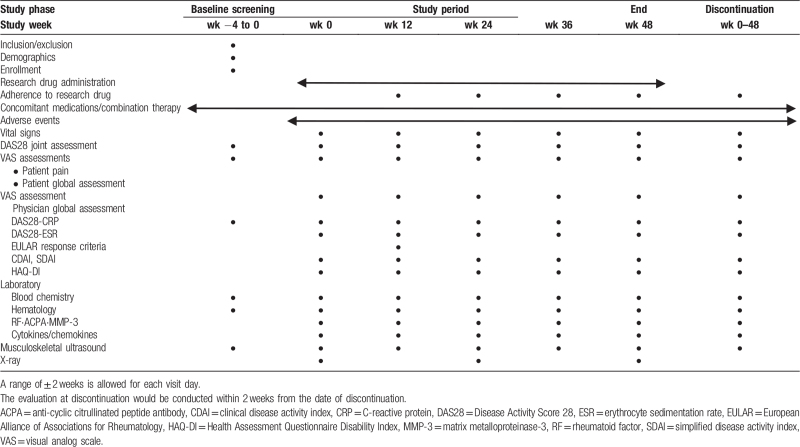
Treatment schedule and outcome measures.

#### 3.4.1 Clinical disease activity

Clinical disease activity will be evaluated by each attending physician (Japan College of Rheumatology-certified rheumatologists) based on the values of the DAS28-ESR, DAS28-CRP score, clinical disease activity index (CDAI), simplified disease activity index (SDAI), and the EULAR response criteria.^[11]^ At each visit, 28 joints, including the bilateral glenohumeral, elbow, wrist, metacarpophalangeal (MCP), interphalangeal, proximal interphalangeal of the hand, and knee joints, will be assessed for tenderness and swelling. Global and pain assessment and evaluator global assessment of each patient will be established on a 0–100-mm visual analog scale, and functional assessment will be scored according to the Health Assessment Questionnaire-Disability Index (HAQ-DI).

#### 3.4.2 MSUS assessments

Participants will undergo imaging by MSUS at baseline and 12, 24, 36, and 48 weeks. Each MSUS examination will be conducted by a Japan College of Rheumatology-certified sonographer. Systematic multiplanar grayscale (GS) and PD examinations of the joints will be performed using a multifrequency linear transducer (12–24 MHz). PD will be used depending on which Doppler modality of the individual machine is the most sensitive. The Doppler settings will be adjusted at each hospital in accordance with published recommendations.^[12,13]^ There will be no change in the MSUS settings during the study and no software upgrade.

Articular synovitis will be assessed by MSUS at dorsal views of 22 joints, including the bilateral wrist joints, 1st–5th MCP joints, interphalangeal joints, and 2nd–5th proximal interphalangeal joints. Each joint will be scored for GS and PD on a scale of 0 to 3 in a semiquantitative manner. The sum of the GS or PD scores will be considered the total GS or PD score, respectively. The Outcome Measures in Rheumatology-EULAR combined PDUS score (i.e., the combined PD score) and Global Outcome Measures in Rheumatology-EULAR Synovitis score will also be assessed.^[14,15]^ The combined PD score will be combined with synovial hypertrophy as indicated by the GS and PD scores.^[14,15]^

#### 3.4.3 X-ray imaging

X-ray imaging of the bilateral hands (posteroanterior view) and feet (anteroposterior view) will be conducted. Joint damage progression will be evaluated based on the van der Heijde-modified total Sharp score (vdH-mTSS) of 16 areas of each hand for erosions and 15 for joint-space narrowing.^[16]^

#### 3.4.4 Biomarker measurements

Patient serum concentrations of the following biomarkers will be measured: rheumatoid factor (RF) using the latex agglutination turbidimetric immunoassay (LZ test “Eiken” RF; Eiken Chemical Co., Ltd., Tokyo, Japan). Anti-cyclic citrullinated peptide antibodies will be measured using a chemiluminescent immunoassay (STACIA MEBLux CCP test, Medical and Biological Laboratories, Aichi, Japan). Matrix metalloproteinase-3 will be measured using latex turbidimetric immunoassay (Panaclear matrix metalloproteinase-3 “Latex”; Sekisui Medical Co., Ltd., Tokyo, Japan). Multiplex cytokine/chemokine bead assays will be performed using diluted serum supernatants and the MILLIPLEX MAP Human Cytokine/Chemokine Magnetic Bead Panel (Merck KGaA, Darmstadt, Germany) and the Bio-Plex Pro Human Cytokine Assays (Bio-Rad Laboratories, Inc., Hercules, CA) and analyzed with a Bio-Plex MAGPIX Multiplex Reader (Bio-Rad Laboratories, Inc.) in accordance with the manufacturers’ instructions.

The cytokines/chemokines for measurement with the bead panel will include interleukin (IL)-1α, IL-1β, IL-1 receptor antagonist, IL-2, IL-4, IL-5, IL-6, IL-7, IL-8, IL-10, IL-12 (p40), IL-12 (p70), IL-13, IL-15, IL-17A, IL-17F, IL-18, IL-22, IL-27, interferon (IFN)-γ, IFN-α2, CXC motif (CXC)L1 (growth-related oncogene), granulocyte-macrophage colony-stimulating factor, granulocyte colony-stimulating factor, C-X3-C motif ligand 1 (fractalkine), flt-3 ligand, fibroblast growth factor-2, eotaxin, epidermal growth factor, vascular endothelial growth factor, platelet-derived growth factor-AA, soluble CD40 ligand, TNF-α, TNF-β, transforming growth factor-α, C-C motif chemokine ligand (CCL)4 (macrophage inflammatory protein [MIP]-1β), CCL3 (MIP-1α), CCL22 (macrophage-derived chemokine), CCL7 (MCP-3), CCL2 (MCP-1), CXCL10 (IFN-γ-inducible protein-10), vascular cell adhesion molecule-1, and intercellular adhesion molecule-1.

### 3.5 Study endpoints

#### 3.5.1 Primary endpoint

The primary endpoint is maintenance of a DAS28-CRP score of ≤3.2 from week 24 to 48 in patients who achieve a DAS28-CRP score of <2.6 at week 24.

#### 3.5.2 Secondary endpoints

The secondary endpoints of this study are as follows: (1) achievement of a DAS28-CRP score of ≤3.2 at weeks 12, 24, and 36; (2) achievement of a DAS28-CRP score of <2.6 at weeks 12, 24, 36, and 48; (3) clinical relapse (DAS28-CRP score of >3.2) at week 48 in patients who achieved a DAS28-CRP score of <2.6 at week 24; (4) achievement of an EULAR-defined moderate response at week 12; (5) changes in DAS28-ESR and DAS28-CRP scores from baseline to weeks 12, 24, 36, and 48; (6) changes in DAS28-ESR and DAS28-CRP scores from week 24 to 36 and 48; (7) changes in CDAI and SDAI values from baseline to weeks 12, 24, 36, and 48; (8) changes in CDAI and SDAI values from week 24 to 36 and 48; (9) achievement of a CDAI of ≤2.8 at weeks 12, 24, 36, and 48; (10) achievement of a SDAI of ≤3.3 at weeks 12, 24, 36, and 48; (11) changes in biomarker serum levels from baseline to weeks 12, 24, 36, and 48; (12) changes in biomarker serum levels from week 24 to 36 and 48; (13) changes in total PD and GS scores and combined PD score from baseline to weeks 12, 24, 36, and 48; (14) changes in total PD and GS scores and combined PD score from week 24 to 36 and 48; (15) change in vdH-mTSS from baseline to weeks 24 and 48; (16) change in vdH-mTSS from week 24 to 48; (17) change in the HAQ-DI data from baseline to weeks 12, 24, 36, and 48; and (18) change in the HAQ-DI data from week 24 to 36 and 48.

#### 3.5.3 Exploratory endpoints

For further research, the following exploratory endpoints will be assessed:

1. DAS28-ESR and DAS28-CRP scores at baseline and weeks 12, 24, 36, and 48
2. Patient pain visual analog scale score at baseline and weeks 12, 24, 36, and 48
3. Total PD and GS scores and combined PD score at baseline and weeks 12, 24, 36, and 48
4. VdH-mTSS at baseline and weeks 24 and 48
5. HAQ-DI at baseline and weeks 12, 24, 36, and 48
6. Biomarker serum levels at baseline and weeks 12, 24, 36, and 48

### 3.6 AEs

All AEs that occur between the baseline visit and the end of week 48 will be recorded. If necessary, the investigators will administer appropriate treatments. A serious AE is defined as any adverse reaction resulting in any of the following outcomes: life-threatening condition or death; a condition that requires inpatient hospitalization or prolongation of existing hospitalization; and any condition threatening to cause disability or disability, congenital anomaly, or birth defect. All serious AEs will be documented in the medical records and reported to the CRB by the responsible investigator in accordance with Japanese regulations.

### 3.7 Data collection and management

In this study, the data of each patient will be entered into an online, web-based electronic data-capture (EDC) system in the form of an electronic case report (e-CRF). The data of biomarkers, such as cytokines, reassessment MSUS results, and vdH-mTSS results will be collected as external data in electronic data format (e.g., Excel or comma-separated values), rather than entered into the EDC. According to the schedule presented in Table 1, the investigator will collect data at each patient visit during the study period. Appropriate and authorized persons (investigators, clinical trial physicians, and clinical trial collaborators) will be provided access to the EDC system in order to enter and modify the collected patient data. All data recorded in the e-CRF will be checked for consistency with the original materials.

All study findings and documents will be regarded as confidential. Patients will be identified on the e-CRF by an anonymous number, not by name. The confidentiality of all documents that identify the patient will be maintained by the investigator to ensure patient anonymity. During the study, a sponsor-investigator will perform regular site visits to review protocol compliance, conduct source data verification, assess drug accountability and management, and ensure that the study is being conducted according to pertinent regulatory and protocol requirements.

### 3.8 Sample size

First, the predicted point estimate and 95% confidence interval (CI) of the primary endpoint is 0.65 with a half-width of 10% of the 95% CI in the ORAL-Shift trial.^[17]^ Variability of the estimate, one-sided width 95% CI, is clinically valid. The sample size required at the start of upadacitinib monotherapy was calculated as 84. Next, in consideration that 60% of patients achieved a DAS28-CRP score < 2.6 with upadacitinib plus MTX combination therapy in the SELECT-COMPARE^[4]^ and SELECT-SUNRISE^[18]^ trials, the sample size necessary at the start of upadacitinib plus MTX combination therapy was calculated as 140. Here, the candidate predicted point estimates were calculated as 0.4 and 0.7 for the SELECT-COMPARE and SELECT-SUNRISE trials, respectively. The cohort of the SELECT-COMPARE trial was non-Japanese, while that of the SELECT-SUNRISE trial was Japanese. The majority, if not all, of the patients in the proposed cohort will be Japanese. The cohort of the proposed cohort is planned to be similar to that of the SELECT-SUNRISE trail. Because the cohorts will not likely be completely comparable, the effect was considered more conservatively than in the SELECT-SUNRISE trail, as the point estimate of the proposed study is 0.60. Finally, assuming that the dropout rate over the entire study period will be 10%, the target sample size was calculated as 155.

### 3.9 Statistical analysis

The primary analysis is planned to estimate the 95% CI of the primary endpoint (section 3.5.1) via Wilson's score interval.^[12]^ The primary endpoint will be evaluated from a set of participants who achieved a DAS28-CRP score of <2.6 during upadacitinib plus MTX combination therapy (Stage I). The primary analysis will control for type I errors at the 5% significance level. To support the clinical interpretation of the results of the primary analysis, participant-wise time courses of the DAS28-ESR and DAS28-CRP scores will be plotted. The distribution of changes in the total PD and GS scores and the combined PD scores will be summarized as histograms. Other statistical analyses are planned to explore the relationships between the measured results obtained as exploratory outcomes, as detailed in section 3.5.3. All other analyses will be considered as exploratory and will not be adjusted for multiplicity.

## 4 Discussion

The main purpose of this clinical trial is to determine whether clinical relapse of RA can be avoided after discontinuation of MTX in patients who achieved clinical remission with upadacitinib plus MTX combination therapy.

The introduction of JAK inhibitors and bDMARDs has dramatically improved the management of a relatively large number of RA patients.^[3]^ In addition, several studies have evaluated changes in disease activity after discontinuation of MTX in RA patients who achieved a good response to MTX plus bDMARDs or JAK inhibitors.^[17,19–21]^ The ORAL-Shift trial showed that tofacitinib monotherapy after discontinuation of MTX was not inferior to continued tofacitinib plus MTX combination therapy, as determined by changes in the DAS28-ESR scores of RA patients who achieved low disease activity with tofacitinib plus MTX.^[17]^ However, no clinical study has yet evaluated disease activity using MSUS after discontinuation of MTX in RA patients who achieved clinical remission during treatment with MTX plus bDMARDs or JAK inhibitors.

Thus, the strength of this study is the prospective evaluation of treatment effects using MSUS, which can accurately and objectively assess disease activity at the joint level as well as the clinical disease activity indices. In addition, the serum levels of many biomarkers, including cytokines and chemokines, will be broadly analyzed, and parameters at baseline and 24 weeks will be assessed to predict non-relapse after discontinuation of MTX by integrating multilateral assessments (i.e., clinical disease activity indices, MSUS findings, and serum biomarkers).

We will be able to provide optimized treatment options if we could predict patients who could achieve clinical remission treated with upadacitinib plus MTX and then could discontinue MTX without a relapse, from the results of this study.

## Acknowledgments

The author thank our colleagues and staff at the Rheumatology Department of Nagasaki University Hospital for their support.

## Author contributions

All authors have given their final approval of the manuscript to be published as presented.

**Conceptualization:** Toshimasa Shimizu, Shin-ya Kawashiri, Shuntaro Sato, Hiroshi Yamamoto, Atsushi Kawakami.

**Formal analysis:** Shin-ya Kawashiri, Toshimasa Shimizu, Shuntaro Sato, Shimpei Morimoto.

**Investigation:** Shin-ya Kawashiri, Toshimasa Shimizu, Atsushi Kawakami.

**Methodology:** Shin-ya Kawashiri, Toshimasa Shimizu, Shuntaro Sato, Shimpei Morimoto, Hiroshi Yamamoto, Atsushi Kawakami.

**Project administration:** Shin-ya Kawashiri, Toshimasa Shimizu, Yasuko Ito, Yurika Kawazoe.

**Writing – original draft:** Toshimasa Shimizu, Shin-ya Kawashiri.

**Writing – review & editing:** Toshimasa Shimizu, Shin-ya Kawashiri, Shuntaro Sato, Yasuko Ito, Yurika Kawazoe, Rina Kawasaki, Shohei Kuroda, Atsushi Kawakami.
